# Prenatal Hyperandrogenization Induces Metabolic and Endocrine Alterations Which Depend on the Levels of Testosterone Exposure

**DOI:** 10.1371/journal.pone.0037658

**Published:** 2012-05-24

**Authors:** Sabrina Amalfi, Leandro Martín Velez, María Florencia Heber, Susana Vighi, Silvana Rocío Ferreira, Adriana Vega Orozco, Omar Pignataro, Alicia Beatriz Motta

**Affiliations:** 1 Laboratorio de Fisio-patología Ovárica, Centro de Estudios Farmacológicos y Botánicos, Consejo Nacional de Investigaciones Científicas y Técnicas, Universidad de Buenos Aires, Buenos Aires, Argentina; 2 Departamento de Patología, Hospital de Clínicas, Buenos Aires, Argentina; 3 Laboratorio de Investigaciones Biomédicas, Instituto de Biología y Medicina de Cuyo, Buenos Aires, Argentina; 4 Laboratorio de Endocrinología Molecular y Transducción de Señales, Instituto de Biología y Medicina Experimental, Nacional de Investigaciones Científicas y Técnicas, Buenos Aries, Argentina; Universitat de Barcelona, Spain

## Abstract

Prenatal hyperandrogenism is able to induce polycystic ovary syndrome (PCOS) in rats. The aim of the present study was to establish if the levels of prenatal testosterone may determine the extent of metabolic and endocrine alterations during the adult life. Pregnant Sprague Dawley rats were prenatally injected with either 2 or 5 mg free testosterone (groups T2 and T5 respectively) from day 16 to day 19 day of gestation. Female offspring from T2 and T5 displayed different phenotype of PCOS during adult life. Offspring from T2 showed hyperandrogenism, ovarian cysts and ovulatory cycles whereas those from T5 displayed hyperandrogenism, ovarian cysts and anovulatory cycles. Both group showed increased circulating glucose levels after the intraperitoneal glucose tolerance test (IPGTT; an evaluation of insulin resistance). IPGTT was higher in T5 rats and directly correlated with body weight at prepubertal age. However, the decrease in the body weight at prepubertal age was compensated during adult life. Although both groups showed enhanced ovarian steroidogenesis, it appears that the molecular mechanisms involved were different. The higher dose of testosterone enhanced the expression of both the protein that regulates cholesterol availability (the steroidogenic acute regulatory protein (StAR)) and the protein expression of the transcriptional factor: peroxisome proliferator-activated receptor gamma (PPAR gamma). Prenatal hyperandrogenization induced an anti-oxidant response that prevented a possible pro-oxidant status. The higher dose of testosterone induced a pro-inflammatory state in ovarian tissue mediated by increased levels of prostaglandin E (PG) and the protein expression of cyclooxygenase 2 (COX2, the limiting enzyme of PGs synthesis). In summary, our data show that the levels of testosterone prenatally injected modulate the uterine environment and that this, in turn, would be responsible for the endocrine and metabolic abnormalities and the phenotype of PCOS during the adult life.

## Introduction

Polycystic ovary syndrome (PCOS), one of the most common reproductive disorders, affects between 8 to 12% women in their reproductive ages [1]. Women with PCOS display oligo or anovulation, hyperandrogenism and/or ovarian cysts [2]. PCOS is frequently associated with hyperinsulinaemia, insulin resistance syndrome, increased cardiovascular risk and type 2 diabetes mellitus [3]–[5]. Its etiology remains uncertain, but current theories emphasize genetic and intrauterine origins coupled with environmental factors such as diet and altered lifestyle patterns [3].

A battery of animal models used for the study of PCOS has allowed focusing on different aspects of the pathology. In that context, prenatal androgen exposure is able to induce PCOS and metabolic syndrome in adult female rats [6]–[8], monkeys [9] and sheep [10]–[12]. In the brain, a prenatal excess of testosterone induces sex differences [9] and defeminization by increasing pulses of gonadotropin-releasing hormone (GnRH) [13]. In addition, a prenatal excess of testosterone increases body weight, induces insulin resistance [8] and deficiency of 21-hydroxylase [14] during the adult life. Recently, Legro et al [15] reported a direct association between birth weight and metabolic phenotypes in women with PCOS. However, data concerning the mechanisms involved in the prenatal excess of androgen and change in the secretion of hormones is still controversial [8], [13], [16].

Although it is well accepted that prenatally androgenized animal models help in investigating the etiology of PCOS, little is known about whether the androgen concentration of androgen determines the phenotype of PCOS during the adult life. Then, the aim of the present study was to determine whether different doses of testosterone at fetal life have different life-long effects.

Prostaglandins (PGs) modulate different ovarian functions, such as the rupture of ovarian follicles associated with ovulation [17], [18] and luteolysis [19], [20]. In addition, PGE is increased in patients with PCOS [21]. We have previously reported that hyperandrogenization with dehydroepiandrosterone induces a pro-inflammatory status mediated by the PG system in mice [22], [23]. For these reasons we were interested in studying whether different levels of prenatal exposure of testosterone induced a pro-inflammatory status mediated by the PG system in ovarian tissue from adult rats.

**Figure 1:**
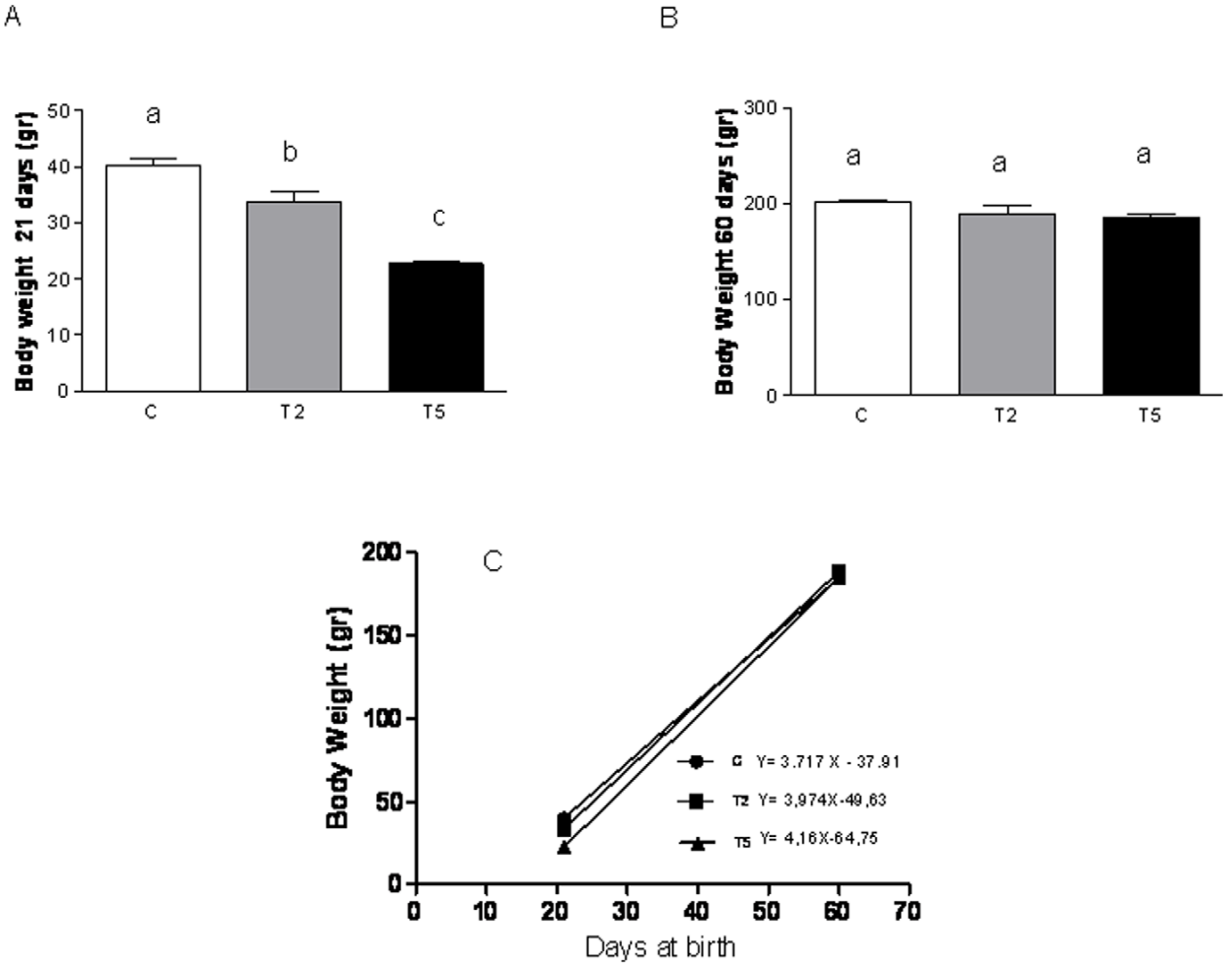
Prenatal hyperandrogenization and body weight. Pregnant Sprague Dawley rats were subcutaneous injected with 2 or 5 mg free testosterone from 16 to 19 day of pregnancy. Female offspring prenatally injected with 2 mg testosterone (T2 group), 5 mg testosterone (T5) or vehicle (C). Panel (A) represents the weight at 21 days of age and panel (B) represents the weight at 60 days of age. Panel (C) represents the growth rates of three groups control, T2 and T5; a vs b P<0.01; b vs c P<0.0001; a vs c P<0.001 by ANOVA test.Each column represents the mean+SEM from ten different animals, N = 20 animals/group.

**Figure 2:**
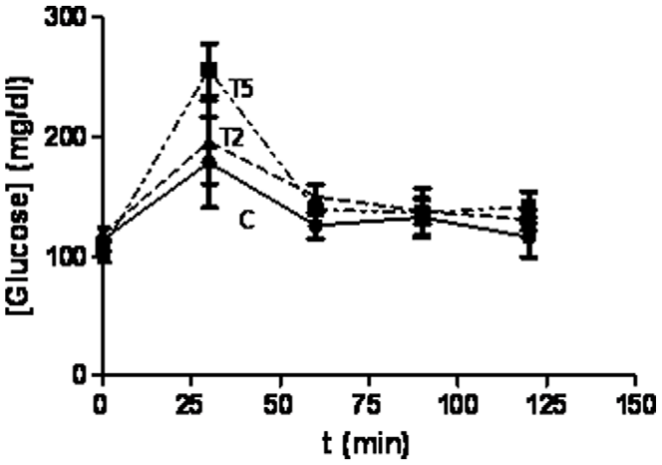
Prenatal hyperandrogenization and glucose homeostasis. As a measurement of glucose homeostasis, dynamic studies were performed in female offspring of Sprague Dawley rats prenatally injected with 2 mg testosterone (T2 group), 5 mg testosterone (T5) or vehicle (C). Blood sample followed by itraperitoneal injection of 2 g dextrose/kg body weight was collected at 0, 30, 60, 90 and 120 post-injection. Each column represents the mean+SEM from ten different animals, N = 20 animals/group.

**Figure 3:**
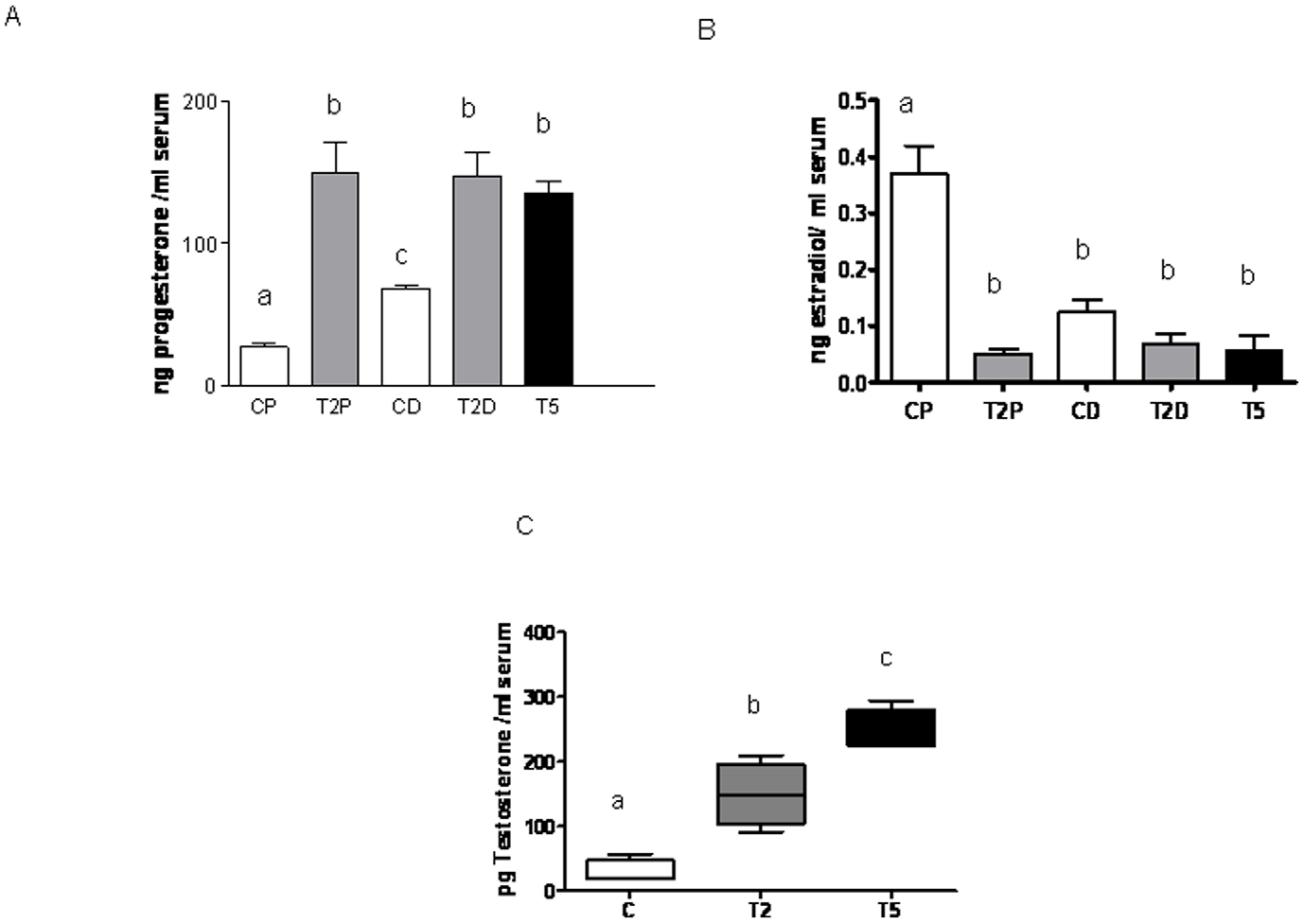
Prenatal hyperandrogenization and ovarian steroidogenesis. (A) Serum progesterone levels, (B) Serum estradiol levels and (C) Serum testosterone levels from female offspring of Sprague Dawley rats prenatally injected with 2 mg testosterone (T2 group), 5 mg testosterone (T5) or vehicle (C). CP: rats from the control group at proestrous stage of the estrous cycle, T2P: rats from the T2 group of treatment at proestrous stage of the estrous cycle CD: rats from the control group at diestrous stage of the estrous cycle, T2D: rats from the T2 group of treatment at diestrous stage of the estrous cycle, T5: rats from the T5 group of treatment. a vs b; a vs c and b vs c P<0.0001 by ANOVA test. Each column represents the mean+SEM from ten different animals, N = 20 animals/group.

**Figure 4:**
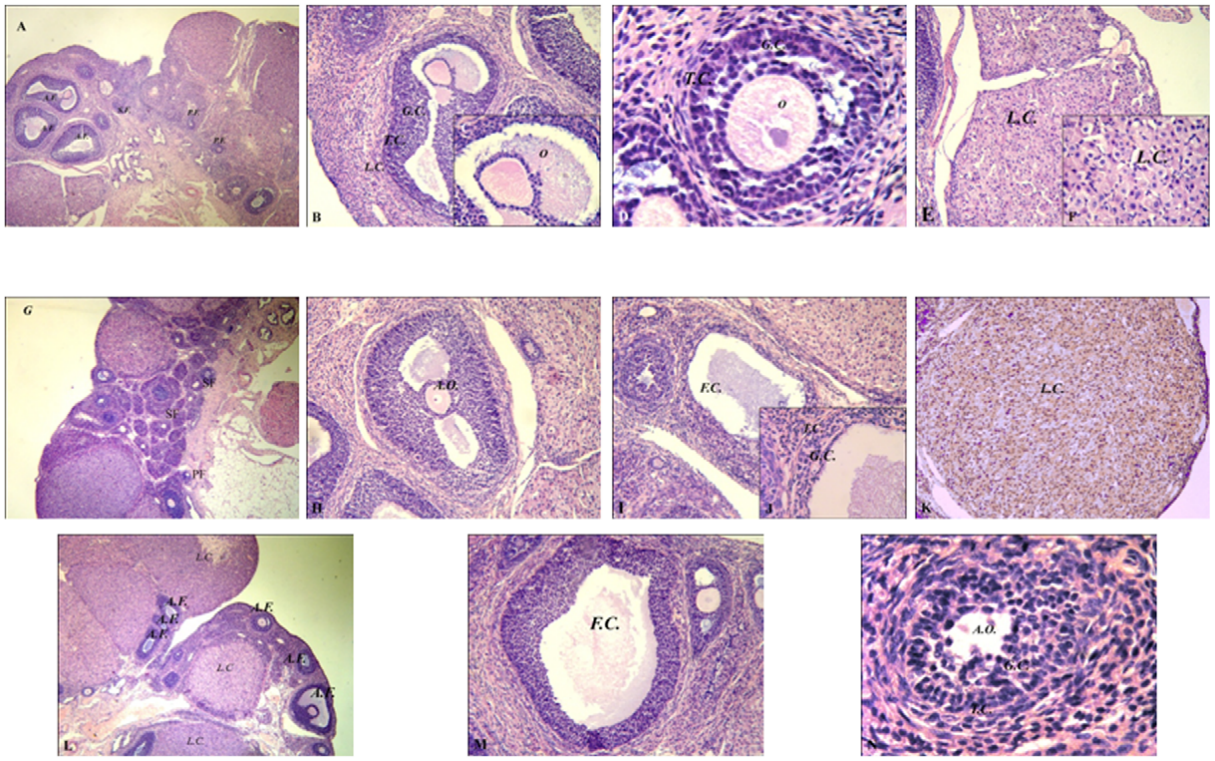
Prenatal hyperandrogenization and ovarian morphology. (A) A representative ovarian tissue section from rats prenatally injected with vehicle (Control group), ×200. (B) Detail of granulosa (GC), theca cells (TC) and luteinized cells (LC) of ovarian tissue from rats prenatally injected with vehicle (Control group), ×400. (C) Magnification of conserved oocyte of ovarian tissue from rats in the Control group, ×1000. (D) Detail of conserved oocytes (O) and distribution of granulosa (GC) and theca cells (TC) of ovarian tissue from rats in the Control group, ×400. (E) Detail of luteinized cells (LC) of ovarian tissue from rats in the Control group, ×400 (F) Magnification of conserved luteinized cells (LC) of ovarian tissue from rats in the Control group, ×1000. (G) A representative ovarian tissue section from rats prenatally injected with 2 mg testosterone (T2 group), PF = primary follicle, SF = secondary follicle, ×400. (H) A representative atretic oocyte (AO) of ovarian tissue from rats in the T2 group, ×400. (I) A representative follicular cyst (FC) of ovarian tissue from rats in the T2 group, ×400. (J) Magnification of granulosa (GC) and theca cell (TC) of the follicular cyst, ×1000. (K) Aspect of luteinized cells (LC) of ovarian tissue from rats in the T2 group, ×1000. (L) A representative section of ovarian tissue from rats prenatally injected with 5 mg testosterone (T5 group), AF = antral follicle, ×200. L.C. = luteal cells (M) Follicular cysts (FC) in ovarian tissue from rats in the T5 group, ×400. (N) A detail of altered organization of granulosa (GC) and theca cells (TC) and atretic oocytes (AO) of ovarian tissue from rats in the T5 group, N = ten ovaries per group.

**Figure 5:**
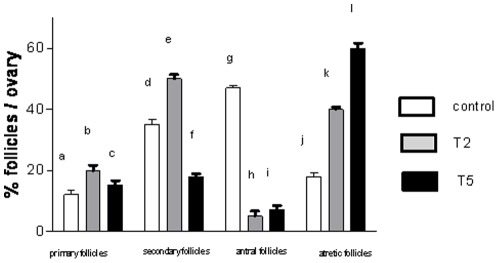
Percentages of follicles (primary, secondary, antral and atretic) per group of treatment: control, T2 and T5. Ten ovaries from each group were fixed in 4% (w/v) paraformaldehyde, included in paraffin, cut 4 µm per section, placed on gelatin-coated slides and stained with haematoxylin and eosin.

**Figure 6:**
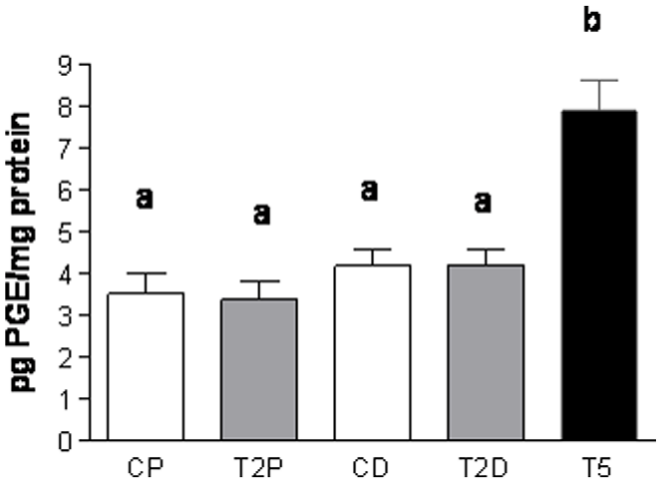
Prenatal hyperandrogenization and ovarian inflammatory status. Ovarian prostaglandin E content from female offspring of Sprague Dawley rats prenatally injected with 2 mg testosterone (T2 group), 5 mg testosterone (T5) or vehicle (C). CP: rats from the control group at proestrous stage of the estrous cycle, T2P: rats from the T2 group of treatment at proestrous stage of the estrous cycle CD: rats from the control group at diestrous stage of the estrous cycle, T2D: rats from the T2 group of treatment at diestrous stage of the estrous cycle, T5: rats from the T5 group of treatment. a vs b P<0.0001 by ANOVA test. Each column represents the mean+SEM from ten different animals, N = 20 animals/group.

**Figure 7:**
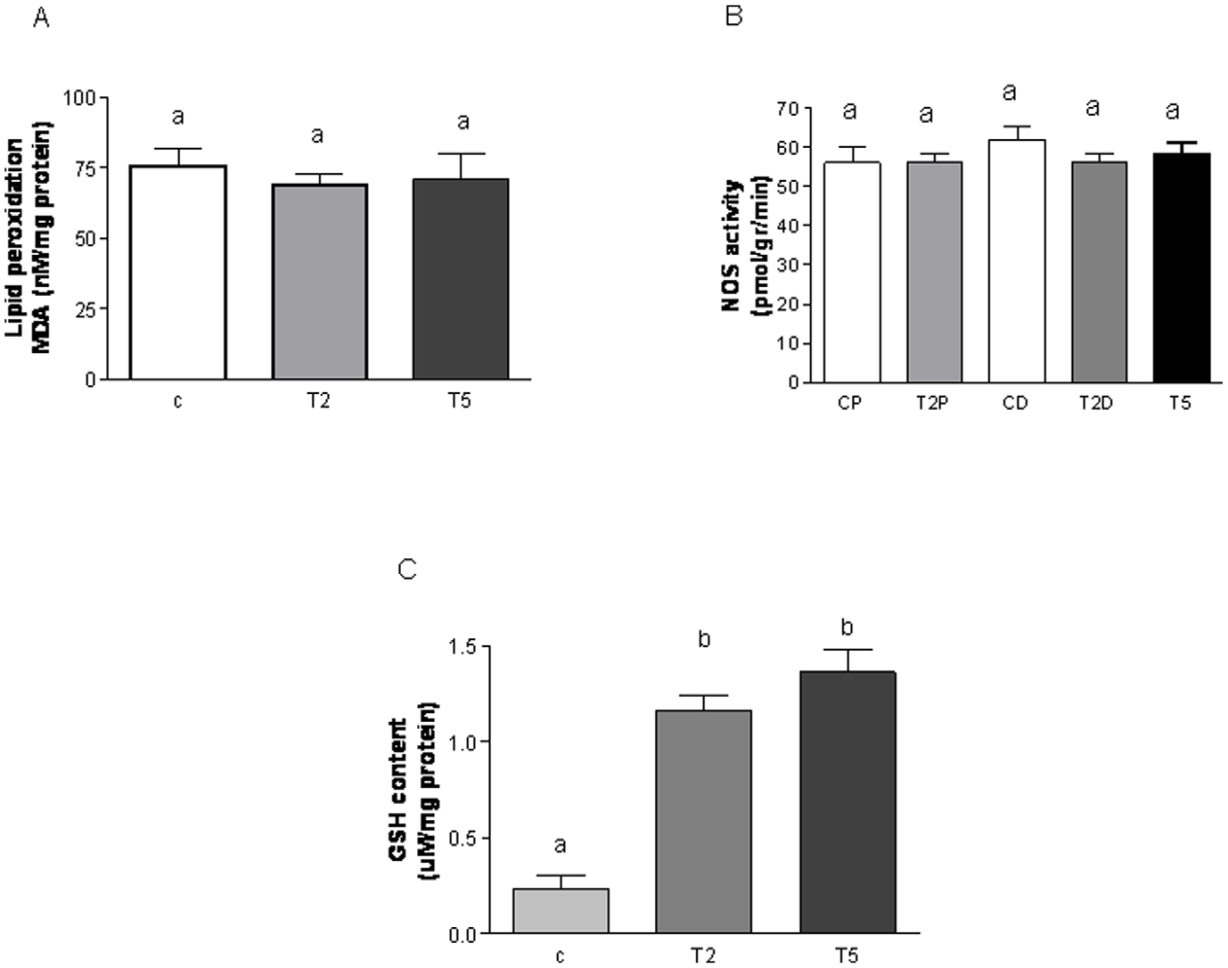
Prenatal hyperandrogenization and ovarian oxidant-antioxidant balance. (A) Lipid peroxidation, (B) Nitric oxide synthase (NOS) activity and (C) Content of anti-oxidant gluthatione (GSH) of ovarian tissue from female offspring of Sprague Dawley rats prenatally injected with 2 mg testosterone (T2 group), 5 mg testosterone (T5) or vehicle (C). CP: rats from the control group at proestrous stage of the estrous cycle, T2P: rats from the T2 group of treatment at proestrous stage of the estrous cycle CD: rats from the control group at diestrous stage of the estrous cycle, T2D: rats from the T2 group of treatment at diestrous stage of the estrous cycle, T5: rats from the T5 group of treatment. a vs b P<0.0001 by ANOVA test. Each column represents the mean+SEM from ten different animals, N = 20 animals/group.

**Figure 8:**
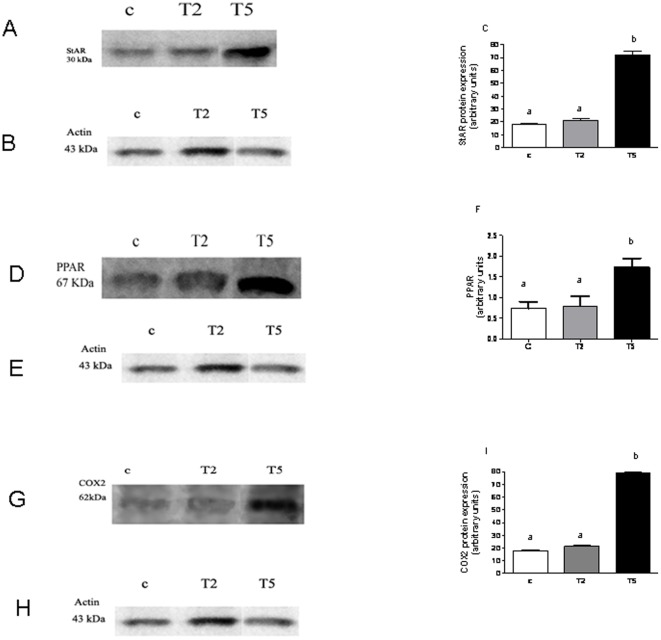
Mechanisms involved in the prenatal hyperandrogenization on ovarian tissue. Western blotting for the expression of the steroidogenic acute regulatory protein (StAR) in ovarian tissue from rats prenatally injected with vehicle (Control group), 2 mg testosterone (T2 group) or 5 mg testosterone (T5 group). Bands correspond to 30 kDa, (A) A representative Western blot, (B) actin as control protein and (C) integrated optical density of the bands. Each column represent mean+SEM of ten different animals. a vs b P<0.0001 by ANOVA test. Western blotting for the expression of the nuclear peroxisome proliferator- activated receptor (PPAR) gamma in ovarian tissue from rats prenatally injected with vehicle (Control group), 2 mg testosterone (T2 group) or 5 mg testosterone (T5 group). Bands correspond to 67 kDa, (D) A representative Western blot, (E) actin as control and (F) integrated optical density of the bands. Each column represent mean+SEM of ten different animals. a vs b P<0.0001 by ANOVA test. Western blotting for the expression of cyclooxygenase 2 (COX2) in ovarian tissue from rats prenatally injected with vehicle (Control group), 2 mg testosterone (T2 group) or 5 mg testosterone (T5 group). Bands correspond to 62 kDa, (G) A representative Western blot, (H) actin as control and (I) integrated optical density of the bands. Each column represent mean+SEM of ten different animals. a vs b P<0.0001 by ANOVA test, N = 20 animals/group.

The excess of androgen induces an imbalance in the ovarian oxidant–antioxidant status characterized by increased production of reactive oxygen species (ROS) [22]–[26]. It has been found that one of the consequences of the increased generation of ROS within ovarian cells is the loss of ovarian function including steroidogenesis [19], [20], [23], [27]. These findings led us to study whether different levels of prenatal excess of testosterone induce ovarian oxidative stress during the adult life.

The nuclear peroxisome proliferator-activated receptor (PPAR) is a family of transcriptional nuclear factors with three isoforms that regulates gene expression [28], [29]. The three PPAR isotypes (alpha, beta and gamma) are detected in developing follicles of several species [29]–[35]. The activation of PPAR gamma regulates the synthesis of steroid hormones in the granulosa cells [36], and the disruption of PPAR gamma in the ovary leads to female subfertility [37]. We have previously reported that acute hyperandrogenization alters the expression of PPAR gamma during early folliculogenesis in rats [38]. For these reasons, here we studied if prenatal hyperandrogenism was able to alter ovarian steroidogenesis through modulating the expression of PPAR gamma. In summary, we studied whether prenatal hyperandrogenism altered endocrine and metabolic pathways during the adult life. We were also interested in establishing if the dose of testosterone to which the fetus was exposed could determine the phenotype of PCOS during the adult life.

## Materials and Methods

### Animals and treatments

Female rats of the Sprague Dawley strain were housed in group cages under controlled conditions of light (12 h light, 12 h dark) and temperature (23–25°C). Animals received food and water *ad libitum*. Virgin female rats were mated with fertile males of the same strain. Day 1 of pregnancy was defined as the morning in which spermatozoa were observed in the vaginal fluid. Between day 16 to day 19 of pregnancy, rats were hyperandrogenized as described before [8]. Briefly, pregnant rats (N = 120) received subcutaneous injections of either 2 mg or 5 mg of free testosterone (T-1500; Sigma, St. Louis, MO) dissolved in 100 µl sesame oil from day 16 to day 19 of pregnancy whereas the control group received only 100 µl of sesame oil. The doses of T2 of free testosterone used result in circulating testosterone levels that are similar to male rats [8], [39]. Under the conditions of our animal facilities, spontaneous term labor occurs on day 22 of gestation. The treatments described did not modify the spontaneous term labor, the female-to-male offspring ratio or the number of pups per litter. Pups were culled from litters to equalize group sizes (ten pups/each mother). Females were separated from males at 21 days of age and randomly chosen. Animals were allowed free access to Purina rat chow and water. All procedures involving animals were conducted in accordance with the Animal Care and Use Committee of Consejo Nacional de Investigaciones Científicas y Técnicas (CONICET) 1996. The Ethic Committee of Facultad de Medicina (UBA) approved the present study. The group of female offspring prenatally treated with 2 mg testosterone was designated as T2, whereas that corresponding to those prenatally treated with 5 mg was designed as group T5 and that without treatment as control group.

### Study protocols

To examine whether prenatal hyperandrogenization altered the intra-uterine development, offspring from animals of the three groups were weighed at 21 and 60 days of age. The offspring were not weight at birth because at that time it was almost impossible to determine reliably the sex of the rats. In addition, the weight at birth has a significant higher error than at 21 days and methodologically it is difficult since manipulation of the offspring could conduce to the rejection of the mother. To establish if prenatal hyperandrogenization induced defeminization, the uro-genital distance was determined at 60 days of age. In order to study whether treatments altered the estrous cycle, the estrous cycle was determined by vaginal smears taken daily from 45 to 60 days of age (day of sacrifice). At 60 days of age, sixty female Sprague Dawley rats per group of treatment were anesthetized with carbon dioxide and killed by decapitation. Trunk blood was collected and serum was separated by centrifugation at 1000 g for 15 min and stored at −80°C until estradiol, progesterone and testosterone were determined by radioimmunoassay (RIA). Ovaries were immediately removed from each group (control, T2 and T5) and divided as follows: 10 were immediately fixed in 4% (w/v) paraformaldehyde for histological studies whereas 40 were immediately frozen at −80°C. Then, 10 of the latter were used for PGE determination by RIA whereas the other 30 used to determine the ovarian oxidant-antioxidant balance (10 ovaries were used to determine the lipid peroxidation index, 10 to determine the nitric oxide synthase activity and 10 ovaries to determine the total glutathione). Other 10 ovaries per group were immediately homogenized in western blotting buffer and stored at −20°C until protein expression of cyclooxygenase 2 (COX2), the limiting enzyme of PGs synthesis, steroidogenic acute regulatory protein (StAR) and PPAR gamma.

### Intraperitoneal glucose tolerance test (IPGTT)

As a measurement of glucose homeostasis and insulin resistance, the intraperitoneal glucose tolerance test (IPGTT) was performed in separate groups of ten animals per group (control, T2 and T5) at 60 days of age after 8 h fast [8]. Briefly, a baseline blood sample was obtained followed by intraperitoneal injection of 2 g/kg body weight dextrose with blood sampling at 30, 60, 90 and 120 min.

Fasting blood glucose was determined by using the Haemoglukotest (Roche) test strips for visual determination in the range of 20–800 mg/100 ml (1–44 mmol/l). The test principle uses the glucose-oxidase/peroxidase reaction. Results are expressed in mg glucose/dl.

### Morphological studies

To study the effect of prenatal hyperandrogenization on the ovarian function, ten ovaries from each group were fixed as described above, were embedded in paraffin wax and consecutively cut. To prevent counting the same follicle twice, 4-µm step sections were mounted at 50-µm intervals onto microscope slides according to the method described by Woodruff et al. [40]. To count the number of different stages of follicles per ovary section, a set of slides was stained with hematoxylin and eosin. An atretic follicle was defined as the follicle that presented more than 10 pycnotic nuclei per follicle; in the smallest follicles, the criterion for atresia was a degenerate oocyte, precocious antrum formation, or both [41], [42]. For morphological analysis, the sections were chosen as follows: five from each extreme and five from the middle of each ovary. Five ovaries from each group were observed for three different researchers.

### Progesterone and estradiol radioimmunoassay (RIA)

Serum progesterone and estradiol levels were determined by specific RIA as described before [19]. Briefly, serum samples from ten rats per treatment were extracted with the same volume of diethyl ether three times. The extracts were collected and evaporated in a vacuum oven and saved at −80°C until the RIA was performed. The antibodies from progesterone and estradiol were provided by Dr Niswender (Colorado State University, Fort Collins, CO, USA). Both sensitivities were 5–10 pg/tube, 2–5 µl of serum. Results are expressed as ng progesterone or estradiol/ml serum.

### Testosterone radioimmunoassay (RIA)

Testosterone was quantified by RIA as previously described [43], [44]. Briefly, serum samples were extracted as described for progesterone and estradiol. The utility range of the assay was 25–1600 pg. The intra-assay and inter-assay variations were 7.5 and 15.1%, respectively. Results are expressed as pg testosterone/ml serum.

### Prostaglandin radioimmunoassay (RIA)

To study whether prenatal hyperandrogenism was able to induce a pro-inflamatory state in ovarian tissue, PGE was determined by RIA as previously reported [19]. Briefly, one ovary per point (ten points per treatment) was weighed and homogenized three times in ethylic alcohol 1∶5 (weight of tissue: volume of alcohol) at room temperature. The extracts were collected and evaporated in a vacuum oven and saved at −80°C until the RIA was performed. PGE was quantified by using rabbit antiserum from Sigma Chemical Co. Sensitivity was 10 pg/tube and cross-reactivity with other PGs was <0.1%. Results are expressed as pg/mg tissue.

### Oxidative stress-related parameters

#### Lipid peroxidation

The amount of malondialdehyde (MDA) formed from the breakdown of polyunsaturated fatty acids may be taken as an index of peroxidation reaction. The method used in the present study was as previously described [27] and quantifies MDA as the product of lipid peroxidation that reacts with trichloracetic acid–thiobarbituric acid–HCl 163 (15% (w/v); 0.375% (w/v) and 0.25 M, respectively) yielding a red compound that absorbs at 535 nm. Homogenates of ovarian pooled tissue (one ovary per point) were treated with trichloroacetic acid–thiobarbituric acid–HCl and heated for 15 min in boiling water bath. After cooling, the flocculent precipitate was removed by centrifugation at 1000 g for 10 min. The absorbance of samples was determined at 535 nm. Content of thiobarbituric acid reactants were expressed as nM MDA formed/mg protein.

### Ovarian nitric oxide synthase activity

Nitric oxide synthase (NOS) activity was evaluated as a measure of NO produced by ovarian tissue. NOS was quantified by monitoring the production of [L-^14^C] citrulline from [L-^14^C] arginine as described previously [19]. Briefly, the frozen ovarian tissue (one ovary per point and ten points per treatment) was homogenized (Tissuemizer Tekmar; Thomas Scientific, Swedesboro, NJ, USA) at 0°C in three volumes of 50 mM Hepes, 1 mM DL-dithiothreitol, 1 mM NADPH and 50 mM L-valine, pH = 7.5. Samples were incubated at 37°C for 15 min with 10 microM [^14^C] arginine (0.3 microCi; 1 Ci = 37 GBq). The samples were centrifuged for 10 min at 1000 *g* and then applied to 1 ml of DOWEX AG50W-X8 (Na+ form; Bio-Rad, Hercules, CA, USA) resin. The radioactivity was measured by liquid scintillation counting. Results are expressed as pmol/g.min.

### Glutathione (GSH) content

The antioxidant metabolite GSH was quantified as previously described [23]. The reduced form of GSH comprises the bulk of cellular protein sulphydryl groups. Thus, measurement of acid-soluble thiol is used to estimate the GSH content in tissue extracts. Briefly, one ovary per point and ten points for group was homogenized in homogenization buffer (EDTA (1 mM), KCl (150 mM), beta Mercoptoethanol (1 mM), Trizma base (20 mM) and sacarose (500 mM), pH = 7.6) and centrifuged at 800 g for 10 minutes at 4°C. Then, supernatants (50 microl/point) were incubated with 800 microl of 1.5 M Tris buffer (pH 7.4) containing 50 microl of 5.10^−3^ M NADPH and 6 IU of GSH reductase. The reaction involves the enzymatic reduction of the oxidized form (GSSG) to GSH. When Ellman's reagent (a sulphydryl reagent 5, 5-dithiobis-2 nitrobenzoic acid; Sigma and Co, St Louis, MO, USA) is added to the incubation medium, the chromophoric product resulting from this reaction develops a molar absorption at 412 nm that is linear to the first beyond 6 min; after this, the reaction remains constant. Results are expressed as microM GSH/mg protein.

### Western blotting

Ovarian tissue were lysed for 20 min at 4°C in lysis buffer (20 mM Tris–HCl, pH = 8.0, 137 mM NaCl, 1% Nonidet P-40 and 10% glycerol) supplemented with protease inhibitors (0.5 mM PMSF, 0.025 mM N-CBZ-L-phenylalanine chloromethyl ketone, 0.025 mM *N′*-*p*-tosyl-lysine chloromethyl ketone and 0.025 mM L-1-tosylamide-2-phenyl-ethylchloromethyl ketone). The lysate was centrifuged at 4°C for 10 min at 10,000 g and the pellet discarded. Protein concentrations in the supernatant were measured by the Bradford assay (Bio-Rad). After boiling for 5 min, 90 microg of protein from each sample was applied to an SDS-polyacrylamide gel (10%) and electrophoresis was performed at 100 Volts for 1.5 h. The separated proteins were transferred onto nitrocellulose membranes in transfer buffer (20% methanol, vol/vol; 0.19 M glycine; 0.025 M Tris-Base, pH = 8.3) for 1 h at 4°C. Blots were blocked for 1.5 h in TBS (4 mM Tris–HCl, pH = 7.5, 100 mM NaCl) containing bovine serum albumin (0.1%) at room temperature. Rabbit polyclonal anti-COX2 (Santa Cruz Biotechnology, Inc., USA) (1∶200 overnight), rabbit polyclonal anti-StAR (Cayman, Ann Arbor, MI, USA) (1∶2000 overnight) or rabbit polyclonal anti-PPAR gamma (Cayman, Ann Arbor, MI, USA) (1∶2500 overnight) were used as primary antibodies. Rainbow-colored protein mass markers (14.3–200 kDa, Bio-Rad) were applied to samples to determine the bands of COX2 (72 kDa), StAR (30 kDa) and PPAR gamma (67 kDa). Protein bands were visualized by incubating the blots with biotin-conjugated secondary anti-rabbit IgG (1∶5000, 1 h) followed by streptavidin–peroxidase complex and diaminobenzidine solution. Consistency of protein loading was evaluated by staining the membranes with Ponceau-S and applying the protein beta actin (43 kDa) (Sigma Co, USA). The intensities (area×density) of the individual bands on western blots were quantified by densitometry (Model GS-700, Imaging Densitometer, Bio-Rad). The experiment was independently repeated three times. Results are expressed in arbitrary units.

### Protein concentration

Ovarian protein concentration was determined as described by Bradford [45].

### Statistical analysis

Statistical analyses were carried out by using the Instant program (GraphPad software, San Diego, CA, USA). ANOVA followed by Newman- Keuls test were used to compare all pairs of columns. A Bonferroni correction for multiple testing was used to adjust the threshold for statistical significance to P<0.05.

## Results

### Prenatal hyperandrogenization and body weight

In order to determine whether prenatal hyperandrogenization affected fetal development, we determined the body weight at 21 and 60 days of age. We found that hyperandrogenization induces an adverse intrauterine condition since it diminished the body weight at 21 days (Fig. 1A, a vs b P<0.01; b vs c P<0.0001; a vs c P<0.001) as compared with controls. Therefore, the higher dose of androgen injected caused a significant decrease in body weight (Fig. 1; T2 vs T5 P<0.0001). This adverse effect of prenatal hyperandrogenization was compensated when the animals were 60 days of age since no significant differences were found between groups (Fig. 1B). This mechanism of compensation in the body weight was clearly manifested by the slope of the growth curve (Fig. 1C). We also found that the “compensatory mechanism” was directly related to the dose of testosterone prenatally injected, thus, the slope of the growth curve (slope of control group = 3.717; slope of T2 = 3.974; slope of T5 = 4.160) increased (r = 0.99, p = 0.0001) with the dose of testosterone (r = 0.66, p = 0.0001).

### Prenatal hyperandrogenization and glucose homeostasis

The IPGTT used to evaluate homeostasis of glucose. Figure 2 showed that prenatal hyperandrogenization induced increased levels of circulating glucose. We also found that this effect was directly related to the dose of testosterone prenatally injected. The area under the curve (control = 16557±200; T2 = 18225±150; T5 = 19638±130 arbitrary units) of glucose concentration clearly shows that circulating glucose was significantly higher in T5 than in T2 (P = 0.0001, r = 0.999).

### Glucose homeostasis and body weight

Increased circulating glucose after IPGTT directly correlated (r = 0.99, p = 0.0001) with the slope of the growth curve (r = 0.66, p = 0.0001).

### Prenatal hyperandrogenization and estrous cycle

The uro-genital distance (UGD) determined at 60 days of age showed that prenatal hyperandrogenization induced defeminization since the UGD was significantly increased in T2 and T5 (control = 1.36±0.15; T2 = 1.66±0.20; T5 = 1.75±0.08 cm; T2 vs control P<0.05; T2 vs T5 P<0.001). With respect to the sexual cycle, we found that 80/80 (100%) of control rats showed a regular estrous cycle (4–6 days). In contrast, only 16/80 (20%) of rats from T2, showed a regular estrous cycle (4–6 days) and T5 rats showed vaginal opening atresia. Vaginal smears from rats from T2 group which did not ovulate showed that these rats stayed at diestrous stage.

### Prenatal hyperandrogenization and Ovarian Steroidogenesis

Since hormonal levels change through the estrous cycle, we determined both serum progesterone and estradiol levels in two well differentiated stages of the cycle: proestrus and diestrus. We chose diestrus because rats from T2 which did not ovulate stayed at that stage and we chose proestrus because it is the stage of follicular development which is one of the altered conditions in PCOS. We found that T2 rats showed increased serum progesterone levels as compared with controls in both proestrus and diestrus stages without significant differences between them (Fig. 3A; a vs b; a vs c and b vs c P<0.0001). Similarly, T5 rats showed significantly increased serum progesterone levels as compared to controls. No differences were found between rats from T2 and T5 (Fig. 3A). As expected, serum levels of estradiol were lower in control rats in diestrus than in control rats in proestrus (Fig. 3B; P<0.0001). T2 and T5 showed decreased levels of serum estradiol as compared with control rats in proestrus (Fig. 3B; P<0.0001). Serum testosterone levels were increased in both T2 and T5 as compared with controls (Fig. 3C; P<0.0001). Rats from T5 showed higher serum testosterone levels than rats from T2 (Fig. 3C; P<0.0001).

### Prenatal hyperandrogenization and ovarian morphology

Ovaries from T2 rats (Fig. 4A–K) and ovaries from T5 rats (Fig. 4L–O) were smaller than controls (control = 55±9 mg vs T2 = 38±5 mg; P<0.005; control = 55±9 mg vs T5 = 42±6 mg, P<0.005). Ovaries from T2 rats had a larger number of developing follicles in early stages (primary and secondary follicles: PF and SF respectively) than controls. These data were quantified in Fig. 5: b vs a and e vs d; P<0.0001. Ovaries from T2 rats showed smaller number of antral follicles (Fig. 4G) than ovaries from control rats (Fig. 4A). These data were quantified in Fig. 5: h vs g P<0.0001. In contrast with the oocytes from control rats (Fig. 4C and 4D), oocytes from T2 rats suffered atresia (Fig. 4H) that it was quantified in Fig. 5: k vs j P<0.0001, and displayed follicular cysts (three cysts/ovary) (Fig. 4I) with altered distribution of granulosa (GC) and theca (TC) cells (Fig. 4 J) as compared with control ovaries (Fig. 4B and 4D). Finally, ovaries from T2 rats displayed abnormal hyper-luteinization (Fig. 4K) characterized by enhanced pyknotic cells with compacted chromatin (Fig. 4K) as compared with controls (Fig. 4E and 4F).

Ovaries from T5 rats showed an increased number of atretic follicles (Fig. 4L: AtF) as compared with controls (Fig. 4A). Since rats from T5 group showed vaginal opening atresia, we used the ovarian morphology to compare ovarian structures with the other groups. Ovarian morphology of rats from T5 group (Fig. 4L) was similar to that of rats from the T2 group which did not ovulate and the two groups stayed in constant diestrus. These data were quantified in Fig. 5: l vs j P<0.0001, and follicular cysts (three cysts per ovary) (Fig. 4M: FC: follicular cysts). Oocytes from ovaries from T5 rats showed atresia (Fig. 4N: AO) and a disorganized distribution of the surrounding layers of granulosa (GC) and theca (TC) cells (Fig. 4N). The number of corpus luteum per ovary between groups did not differ (five corpus luteum/ovary) (data not shown).

### Prenatal hyperandrogenization and ovarian inflammatory status

To assess whether prenatal hyperandrogenization was able to induce a pro-inflammatory status in the ovarian tissue, we determined the PGE content. In order to establish whether the estrous cycle is able to modify the PGE content, we quantified PGE from ovarian tissue in proestrus and diestrus (Fig. 6; P<0.0001). We found that prenatal T2 hyperandrogenization did not modify PGE content in either stage but that prenatal T5 hyperandrogenization significantly increased PGE content as compared with controls and T2 rats (Fig. 6; P<0.0001).

### Prenatal hyperandrogenization and ovarian oxidant-antioxidant balance

To determine whether prenatal hyperandrogenization was able to induce ovarian oxidative stress, lipid peroxidation index, nitric oxide synthase activity and the antioxidant metabolite; GSH were evaluated in the ovarian tissue from control, T2 and T5 rats. Since the content of MDA (the main product which results of lipid peroxidation) was not modified either in ovarian tissue from T2 or T5 rats, we inferred no damage in the ovarian membrane (Fig. 7A). To evaluate the possible accumulation of ROS, we quantified NOS activity. We found that NOS activity was not modified in T2 or T5 (Fig. 7B). However, the ovarian content of the antioxidant metabolite GSH was increased in ovarian tissues from T2 and T5 rats (Fig. 7C; P<0.0001).

### Mechanisms involved in the prenatal hyperandrogenization in ovarian tissue

To clarify some of the molecular mechanisms involved in the altered ovarian steroidogenesis induced by prenatal hyperandrogenization, the protein expression of the limiting protein that regulates cholesterol availability, the steroidogenic acute regulatory protein: StAR and the protein expression of the transcriptional factor PPAR gamma were evaluated by Western blotting (Fig. 8; P<0.0001).

We found that the protein expression of StAR was increased in ovarian tissue from T5 rats as compared to both controls and T2 rats (Fig. 8A–C; P<0.0001). We also found that the protein expression of PPAR gamma was increased in ovarian tissue from T5 rats as compared to both controls and T2 rats (Fig. 8D–F; P<0.0001).

In order to determine whether protein expression of COX2, the limiting enzyme of PG synthesis, was affected by prenatal hyperandrogenization, we evaluated the protein expression of COX2. We found that protein expression of COX2 was increased in ovarian tissue from T5 rats as compared with controls (Fig. 8G–I; P<0.0001). No significant differences were found between T2 rats and controls (Fig. 8G–I; P<0.0001).

A direct correlation was found between COX2 (T5 vs T2 and control, P = 0.0001, r = 0.999) and PPAR gamma (T5 vs T2 and control, P = 0.01; r = 0.950).

## Discussion

Given the limitations in human studies, murine models are an important tool to study PCOS. Prenatal hyperandrogenism is considered as one of the best animal models since, it allows studying fetal programming [46], [47]. In that context, there is agreement that rats have to be prenatally hyperandrogenized from days 16 to 19 of gestation (which represent the post- placentation time and the period in which androgens are responsible for neuromodulations). There is also agreement that the dose of androgen has to be similar to the levels observed in male rats, however, the dose to be injected remains controversial. Wu et al [16] reported that doses of free testosterone large than 3 mg result in mortality of fetuses. These authors also found that female offspring were entirely defeminized. However, Demissie et al [8] reported that 5 mg testosterone did not induce mortality in fetuses or offspring but induced defeminization. In the present study, we used 2 and 5 mg free testosterone to induce prenatal androgenization and found that neither doses induced mortality in the fetuses or the offspring. Moreover, our data are the first demonstration that alterations induced by prenatal hyperandrogenization depend on the intrauterine dose which in turn would determine the PCOS phenotype during the adult life. Offspring prenatally hyperandrogenized with 2 mg free testosterone (T2 group) showed increased levels of serum testosterone, ovarian cysts, and hyperglycemia but they were able to ovulate, whereas offspring prenatally hyperandrogenized with 5 mg free (T5 group) showed increased levels of serum testosterone, hyperglycemia and ovarian cysts but they stayed in constant diestrus. In fact, it was reflected by the increased levels of progesterone found in animals from T5 group as compared with controls. These findings are in agreement with our previous findings [22] and with other authors [48] and demonstrate that hyperandrogenization induces accelerated follicular growth. As shown by the increased UGD, we also found that both doses of free testosterone induced defeminization. These findings are in agreement with previous reports [13] and suggest that prenatal hyperandrogenism affects the neurosecretory system of gonadotropin-releasing hormone (GnRH). Taken together our findings suggest that not only the timing of the in uterus hyperandrogenization [8], [16] but also the concentration of androgens to which the fetus is subjected would be responsible for endocrine and metabolic alterations that in turn, would define the PCOS phenotype. Four phenotypes of PCOS have been proposed: phenotype 1 or classic defined by anovulation (AO)+biochemical or clinical hyperandrogenism (HA) and ovarian cysts (C), phenotype 2 defined by AO+HA, phenotype 3 defined by HA+ C and phenotype 4 defined by AO+C [4]. However, the differences between them are poorly studied. Recently, Panidis et al [49] reported that insulin resistance defines the PCOS phenotype. In that context, in a prospective study of 1,212 PCOS patients, they reported that phenotype 1 is associated with more insulin resistance and more pronounced hyperandrogenism than the other phenotypes and that phenotypes 2 and 4 are associated not only with insulin resistance but also with obesity.

Here we found that adult rats prenatally treated with 5 mg free testosterone showed features corresponding to phenotype 1 whereas those treated with 2 mg free testosterone displayed an altered condition such as that of phenotype 3. In agreement with Panidis et al. [49], we found that the more severe alterations of the two phenotypes correlated with a more negative impact on glucose homeostasis and consequently in insulin resistance as we assume analyzing the area under the curve of the glucose homeostasis in agreement with previous reports [8]. On the other hand, we demonstrated that the more severe condition of PCOS phenotype (rats from T5 group which correlated to phenotype 1) showed lower body weight at 21 days of age than T2 and control rats. In addition, we found that the lower body weight correlated to the fastest weight gain as manifested by: i) no significant differences in the body weight at 60 days of ages and ii) the highest slope of the growth curve in the lowest body weight. These findings led us to suggest that a high dose of prenatal testosterone could correlate to a worse intrauterine environment and with more effective compensatory mechanism of growth. The relationship between low birth and precocious pubarche remains controversial and it appears that ethnic origin is involved [50]–[57]. However, it is well accepted that there is an association between low birth and endocrine-metabolic abnormalities including hyperinsulinemia, dyslipidemia and ovarian hyperandrogenism [52], [54], [57]. In summary, our results demonstrate that the severe PCOS phenotype induces more endocrine and -metabolic alterations and consequently a high response related to insulin resistance. In fact, Barker et al [58] reported that systolic blood pressure during adult life is inversely related to birth weight. Later studies confirmed a direct association between low birth and insulin resistance during adult life [59]–[61]. All these findings allow characterizing the syndrome X or *small baby syndrome* that postulates that an adverse intrauterine environment results in a rapid response to glucose metabolism characterized by lower insulin sensitivity. Here we demonstrated not only that increased levels of intrauterine testosterone exposure induce severe alterations but also that the mechanisms involved are different. Both prenatal androgenization (T2 and T5 rats) displayed enhanced ovarian steroidogenesis characterized by increased levels of progesterone and testosterone and decreased levels of estradiol. It is important to point out that enhanced ovarian steroidogenesis was characterized by an enhanced luteinization as it can be seen in the morphological studies. These data are in agreement with previous findings about an accelerated steroidogenic capacity of theca cells from women with PCOS [62]. With respect to the mechanism involved, we found that only the higher dose of testosterone was able to increase the protein expression of StAR, suggesting another pathway for the lower dose of prenatal testosterone treatment. A wide range of proteins including luteinizing hormone receptor, insulin hormone receptor, lipoprotein receptor, StAR, P450 side-chain cleavage, 3 beta-hydroxysteroid dehydrogenase and cytochrome P450c17 might be over-expressed in ovaries from PCOS patients [63], [64]. Further studies are being designed to clarify which are the pathways involved in the ovarian steroidogenesis in PCOS from rats in the T2 group.

We have demonstrated that prenatal hyperandrogenism induces a pro-inflammatory status in the ovarian tissue mediated by enhanced ovarian PGE production and COX2 protein expression. These findings are in agreement with previous studies using other murine PCOS models [22], [23]. Only the higher dose of prenatally testosterone injected (T5 rats) established an ovarian pro-inflammatory environment and induced anovulation. Although PGs are essential for ovulation [17], [18], the excess of androgen enhances ovarian PG production and consequently induces anovulation [22]. Moreover, anovulatory women with PCOS display increased production of PGE [21]. We have also previously demonstrated that the pattern of inflammation correlates with an enhanced expression of the nuclear transcriptional factor PPAR gamma. This is in agreement with previous findings about the role of PPAR gamma in modulating ovarian steroidogenesis during a condition of acute hyperandrogenism [38]. PPAR gamma not only regulates the lipid metabolism and lipid profile [65] but also is able to induce a pro-inflammatory status by enhancing the production of adipokines [66]. Then, we could infer that the increased protein expression of PPAR gamma would be indirectly related to the pro-inflammatory status mediated by PGE. Moreover, it has been reported that the PPAR system regulates the synthesis of PGs by modulating COX2, the limiting enzyme of this process [67]. These findings could explain why we found that the protein expression of PPAR gamma and COX2 display a similar pattern.

Considering that the excess of androgen induces oxidative stress [22]–[26] and that this, in turn, modulates ovarian function [19], [20], [23], [27] and the fact that nitrogen reactive species (RNOS) represent more aggressive species than ROS, we also evaluated the lipid index, the production of NO and the levels of the antioxidant GSH in the ovarian tissue. We found that neither lipid peroxidation nor NO synthase activity was modified and GSH content was increased by prenatal hyperandrogenization. In agreement with previous findings [68]–[72], these data could suggest that a “controlled response”, given by the increase of the anti-oxidant metabolite GSH, could avoid the accumulation of ROS resulting in a no modification of lipid peroxidation index. The fact that prenatal hyperandrogenization did not modify the production of ovarian NO levels could suggest that that condition did not generate the accumulation of RNOS. However, studies are designed to clarify these points. In summary, this study demonstrates that the levels of testosterone prenatally injected are able to modulate the PCOS phenotype manifested during the adult life. Both the metabolic and endocrine pathways showed during the adult life also depend on the levels of testosterone prenatally injected.
